# Case report: Multi-antibody–positive myasthenia gravis concomitant myositis associated with thymoma

**DOI:** 10.3389/fimmu.2024.1423547

**Published:** 2024-09-05

**Authors:** Chao Huang, Xuelian Dai, Jiacheng Liu, Yunting Zhang, Bianli Yin, Chao Liu, Xiangyang Ren, Zhihui Duan, Huan Yang

**Affiliations:** ^1^ Department of Neurology, Luoyang Central Hospital Affiliated to Zhengzhou University, Luoyang Cerebrovascular Disease (Stroke) Clinical Medical Research Center, Regional Medical Center for Neurological Diseases of Henan Province, Luoyang, China; ^2^ Department of neurology, Xinxiang Medical University, Xinxiang, China; ^3^ Department of Neurology, Xiangya Hospital, Central South University, Changsha, China

**Keywords:** myasthenia gravis, acetylcholine receptor antibodies, idiopathic inflammatory myopathy, antisynthetase syndrome, thymoma

## Abstract

Myasthenia gravis (MG) and idiopathic inflammatory myopathy (IIM) are autoimmune diseases of the nervous system, and their main clinical manifestation is muscle weakness. The concurrent presence of both conditions in the same patient is clinically rare and easily missed. Here, we report the case of a 74-year-old woman who went to the doctor with fluctuating weakness of the limbs and muscle pain. By analyzing the patient’s history and the results of repeated frequency electrical stimulation, chest computed tomography, thigh muscle magnetic resonance imaging, serum antibody detection, lymph node biopsy, etc., she was finally diagnosed with MG-concomitant IIM with squamous cell carcinoma of the thymus. Acetylcholine receptor antibody, titin antibody, ryanodine receptor antibody, anti–JO-1 antibody, and Ro-52 antibody tests were positive. MG-concomitant IIM is often associated with thymoma. The immunopathology mechanism may be different from that of pure MG or IIM, which needs further research.

## 1 Introduction

Myasthenia gravis (MG) is an autoimmune disease with acquired neuromuscular junction transmission disorder mediated by autoantibodies. Acetylcholine receptor (AChR) antibodies are the most common pathogenic antibody. When both titin and ryanodine receptor (RyR) antibodies are positive, it is often highly suggestive that the patient has thymoma (1–3). Idiopathic inflammatory myopathy (IIM) is a group of acquired immune myopathies that mainly include dermatomyositis, polymyositis, immune-mediated necrotizing myopathy, and sporadic inclusion body myositis (4). Myositis antibodies are currently divided into two categories: myositis-specific autoantibodies (MSAs) and myositis-associated antibodies. Anti–aminoacyl-rRNA synthetase (ARS) series antibodies are the most important MSAs (5). Patients who are positive for ARS antibodies have specific clinical symptoms and are generally diagnosed with antisynthetase syndrome (ASS). The anti–JO-1 antibody–positive rate is the highest in ARS (6). MG can be combined with other autoimmune diseases in patients, but there have been few cases of MG combined with IIM, especially ASS. We report a case of MG-concomitant IIM involving multiple autoimmune antibodies and squamous cell carcinoma of the thymus and discuss some aspects of their associations.

## 2 Case presentation

A 74-year-old woman was admitted to our hospital complaining of progressive and fluctuating weakness of the limbs and muscle pain for 1 month. The patient presented with prominent fatigue and difficulties with lifting her upper limbs, such as when drying clothes or climbing stairs, but without any difficulties with swallowing or chewing and no chest tightness or dyspnea, joint pain, rash, dry mouth, dry eyes, or other accompanying symptoms. All her symptoms fluctuated during the day, with dominant twilight activity. She had a history of hypertension and type 2 diabetes. There was no family history of neurological disorders.

**Figure 1:**
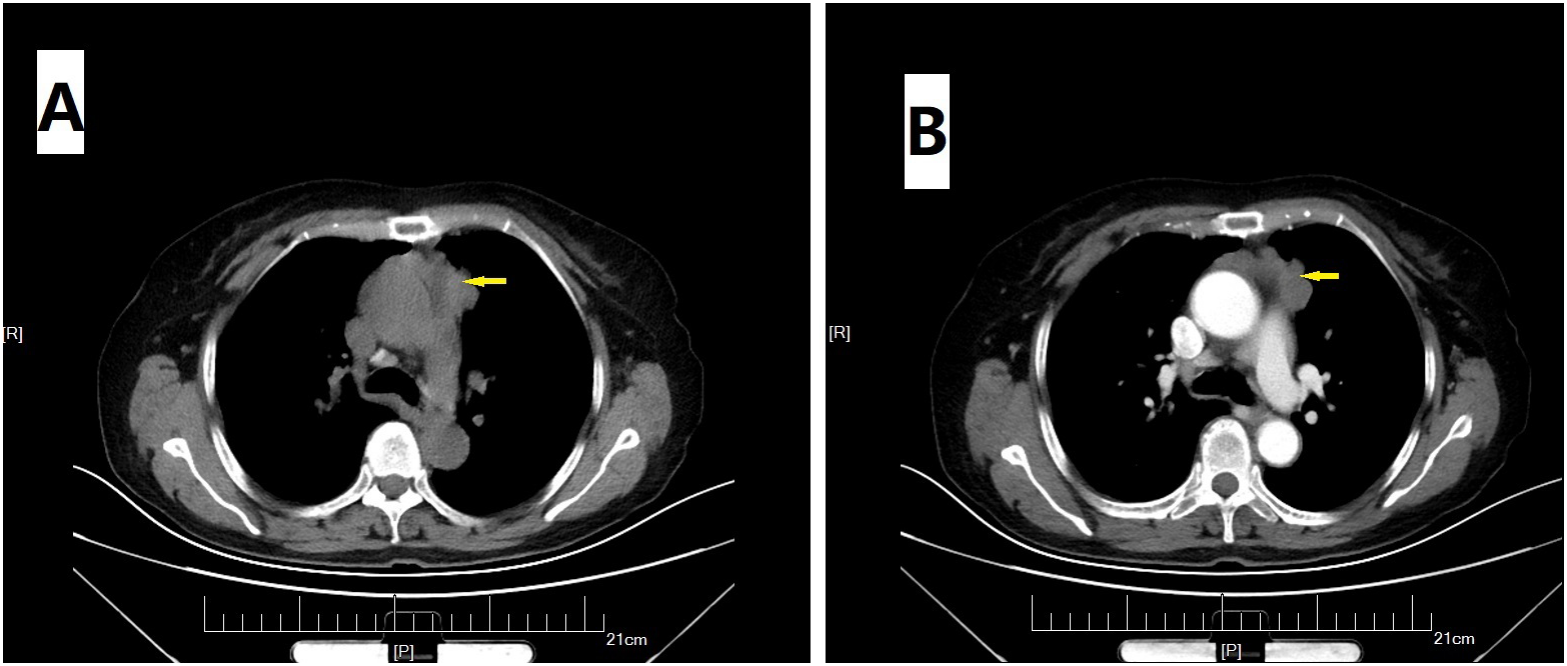
Chest CT scan. Plain **(A)** and enhanced **(B)** images suggested multiple space-occupying lesions (arrow) in the anterior superior mediastinum and left mediastinal pleura.

**Figure 2:**
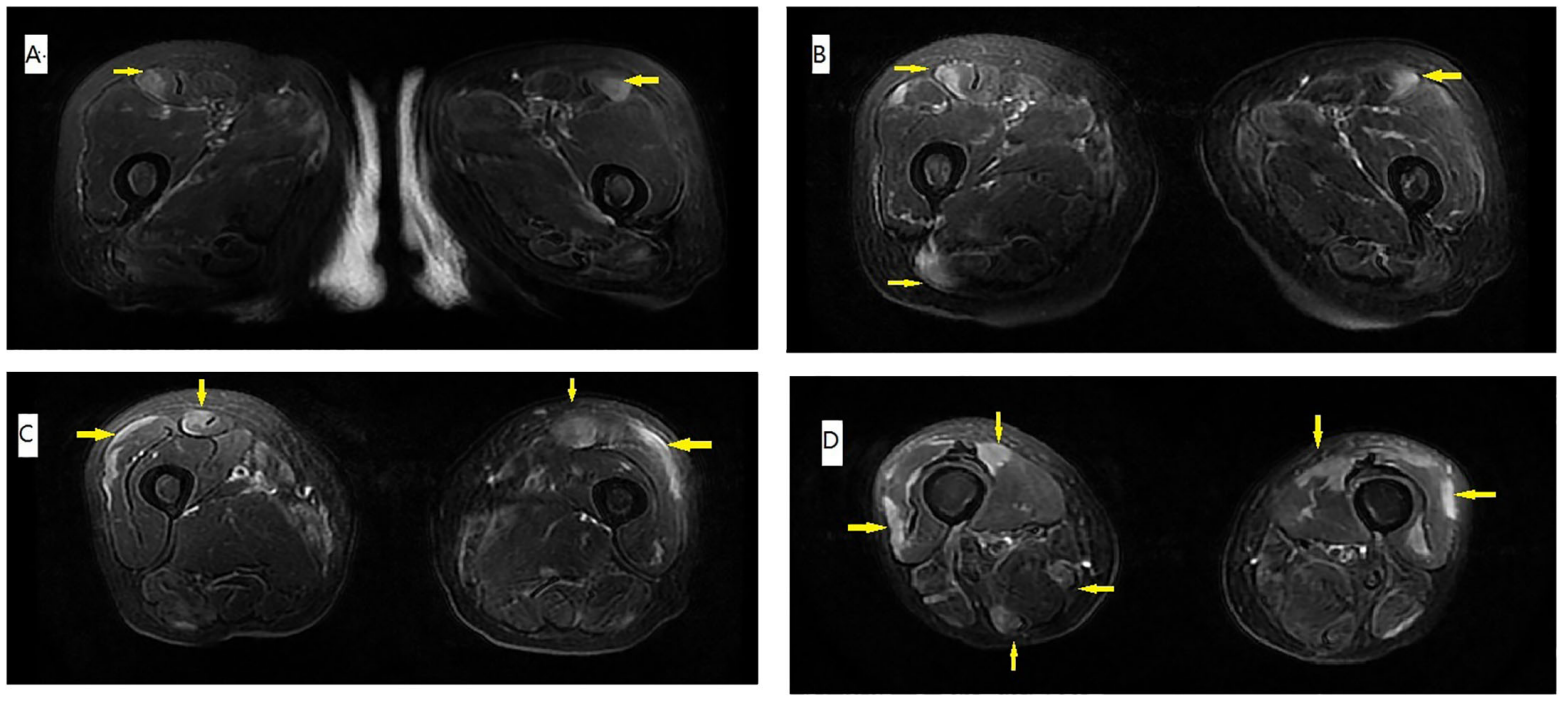
T1 MRI of the thigh. **(A–D)** The muscle edema (arrow) was approximately symmetrical, and the distribution was peripheral and patchy. Signals for gluteus maximus sartorius, rectus femoris, tensor fascia lata, vastus lateralis, gracilis, semimembranosus, and semitendinosus were prominent, compared with the relatively sparse signals for adductor muscles and other muscles of the posterior compartment.

**Figure 3:**
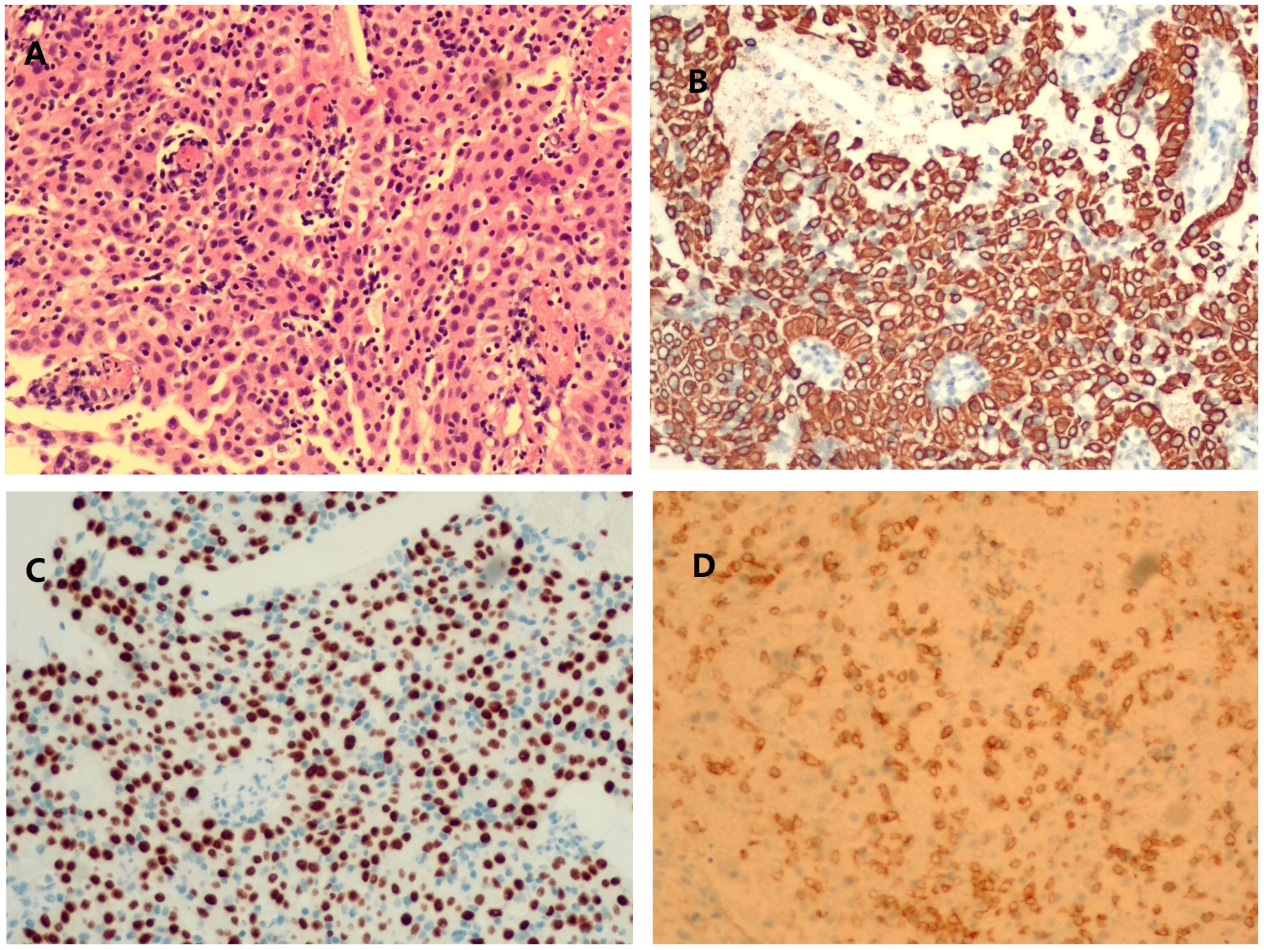
Histopathological images of left cervical lymph node aspiration. hematoxylin and eosin (HE) staining showed an increase in lymphocytes and tumor cells growing in sheets and cords **(A)**. Tissue was positive for cytokeratin staining **(B)**, P40 staining **(C)**, and CD5 staining **(D)**.

On physical examination, distally and proximally accentuated muscle weakness was detected in all extremities (grade 4/5MRC). Laboratory tests showed aspartate transaminase, 37 U/L↑; lactate dehydrogenase, 279 U/L↑; creatine kinase, 206 U/L↑; creatine kinase-myocardial isoenzyme, 39 U/L↑; α-hydroxybutyrate dehydrogenase, 213 U/L↑; total cholesterol, 6.62 mmol/L↑; and D-dimer, 750 ng/mL↑. Her neostigmine test was negative. On electromyogram, left ulnar nerve, facial nerve, and bilateral accessory nerve low-frequency stimulation and left ulnar nerve high-frequency stimulation (10 Hz) all showed attenuation. Chest computed tomography (CT) plain scan with enhancement indicated that there were multiple space-occupying lesions in the anterior superior mediastinum and left mediastinal pleura, and thymic carcinoma was considered. Enlarged lymph nodes were seen in the left cardiophrenic angle and left supraclavicular fossa, and pathological examination was conducted (**Figure 1**). In a thigh magnetic resonance imaging (MRI) scan, the bilateral gluteus maximus, sartorius, rectus femoris, tensor fascia lata, vastus lateralis, gracilis, semimembranosus, and semitendinosus showed abnormal approximately symmetrical signals with long T1 and T2 relaxation times (**Figure 2**). The pathological report on the left supraclavicular fossa lymph node indicated thymic squamous cell carcinoma (**Figure 3**). Antibody detection for MG (Cytometric bead array (CBA) method) showed the patient to be AChR antibody–positive, titin antibody–positive, and RyR antibody–positive. Myositis antibody spectrum tests showed that she was anti–JO-1–positive and anti–RO-52–positive.

The patient was diagnosed with MG-concomitant IIM with squamous cell carcinoma of the thymus. The quantitative myasthenia gravis score (QMGS) was 6 points, which was reduced to 0 after four plasma exchanges, at which time the symptoms of muscle pain were also significantly improved. Polymorphism of the tacrolimus gene was detected as CYP3A5*3*3. After discharge, she was given tacrolimus capsules (1 mg bid), combined with Wuzhi capsules, and blood drug levels were tested 1 week later. The concentration was 5.2 ng/mL, showing that it basically reached the standard level, and standard radiotherapy was started according to the advice of the oncology department. After 6 months of follow-up, her neurological symptoms were significantly relieved, and the patient was able to return to a normal life.

## 3 Discussion and conclusions

In this case, the patient presented with fluctuating limb weakness and pain onset. Repetitive electrical stimulation indicated a high-frequency decrease. Antibody tests for MG showed that she was positive for AChR, titin, and RyR antibodies. Creatine kinase was slightly elevated, and a thigh MRI showed inflammatory edema, whereas the myositis antibody spectrum was anti–JO-1–positive and anti–RO-52–positive. Chest CT plain scan with enhancement indicated possible thymus carcinoma, and, after pathological examination of the lymph nodes, she was diagnosed with thymic squamous cell carcinoma. Therefore, the final diagnosis was MG-concomitant IIM with squamous cell carcinoma of the thymus.

The clinical manifestations of patients with MG complicated with IIM are often atypical and serious. Due to the involvement of lower motor neurons, the major differential diagnosis is Guillain–Barre syndrome in the early stage. The disease can also be combined with MG, and nerve conduction velocity measurement can help localization and differentiation (7). Eighty percent of patients with simple MG start with external ophthalmoplegia that then gradually spreads to other muscle groups (3). ASS in IIM can involve multiple systems, manifesting as myositis, pulmonary interstitial lesions, arthritis, Raynaud’s phenomenon, mechanic hands, etc. (6, 8). In contrast, when MG is combined with IIM, weakness of the limbs is the most common first symptom; the bulbar and respiratory muscles are more prominently involved, and the patient is prone to secondary pulmonary infection and respiratory failure (1, 9). The disease spectrum in patients with anti–JO-1 antibody positivity includes a variety of diseases, mainly ASS, and anti–JO-1 antibodies can also be found in rheumatoid arthritis, interstitial pneumonia with autoimmune features, Sjogren’s syndrome, systemic lupus erythematosus, systemic vasculitis and other connective tissue diseases, malignant tumors, infections, etc. (10). The anti–Ro-52 antibody is a non-specific autoantibody that is common in a variety of autoimmune diseases, such as IIM, systemic lupus erythematosus, Sjogren’s syndrome, and autoimmune liver disease (11). Therefore, when the antibody is positive, attention should be paid to more in-depth examinations to determine whether the patient has other systemic diseases. In this case, limb weakness was the first symptom, which is consistent with the literature. The clinical symptoms were relatively typical, and no other clinical symptoms or positive test indexes were found to suggest dermatitis, interstitial pneumonia, Sjogren’s syndrome, or other autoimmune diseases. Because of the timely diagnosis and treatment, the disease had not yet developed to the bulbar and respiratory muscle stage of involvement.

MRI plays an important role in the diagnosis of IIM and can be used to assess disease activity and severity. MRI of the thighs of patients with IIM can reveal different degrees of muscle edema, whereas patients with ASS tend to have more obvious muscle edema accompanied by fascial edema, and fat replacement and muscle volume are reduced compared with healthy controls. Some studies compared the thigh MRIs of patients with ASS with those of patients with dermatomyositis and immune-mediated necrotizing myopathy and found that myofascial edema was more common in patients with ASS, along with bilateral asymmetry, obvious edema of the tensor fascia lata, and relatively normal adductor muscles. The muscle involvement pattern shown by the MRI of the thigh of this patient is consistent with the characteristics of ASS (12, 13).

Fifty percent of patients with thymoma have no symptoms when diagnosed, whereas the other half of the population may present with paraneoplastic autoimmune/diseases, of which MG is the most common. Approximately 20%–40% of patients with thymoma present with MG symptoms, and 2.8% of patients with thymoma also have IIM. The coexistence of patients with IIM and MG is rare (14). It has been reported in the literature that 77% of patients with MG combined with IIM can also have a thymoma, which is much higher than the proportion of patients with simple MG (15%) (1). A thymus tumor was also found in this patient, and the pathological diagnosis was squamous cell carcinoma of the thymus. The thymus is where T cells differentiate and mature, and these cells can induce autoimmune tolerance and participate in immune regulation in the body. In a thymoma, the structure and microenvironment of the thymus are destroyed, resulting in the dysregulation of T-cell development and selection, leading to immunodeficiency and the emergence of various autoimmune diseases (15). Patients with MG combined with IIM are likely to have accompanying thymic tumors that may be caused by an unknown immune mechanism related to thymoma, which needs further research and exploration.

Compared with patients with pure MG or IIM, patients with MG combined with IIM have different serum immunological characteristics. According to reports, approximately 50%–60% of ocular MG and 85%–90% of patients with generalized MG have AChR antibodies in their serum, and some are positive for anti-striated muscle antibodies (mainly including titin and RyR antibodies) (1). These signs suggest that a patient has a combined thymoma, and the condition is relatively serious. About 75% of patients with IIM are positive for MSAs (4). Statistical analysis shows that (16), when patients with MG have IIM, the AChR antibody positivity rate can reach 81%, which is roughly the same as that of patients with pure MG. The ASA antibody–positive rate can reach 74% in patients with MG combined with IIM, which is much higher than that of patients with pure MG, whereas their MSA positivity rate is only 10%, and the antibodies reported to show as positive are NXP2, PL-7, PM-Scl, P155/140, etc. (17–19). The involvement of JO-1 antibodies has not been reported, and its positivity rate in patients with MG-IIM is much lower than that in patients with IIM alone. Some scholars have speculated that the occurrence of IIM in patients with this type of MG may be related to the presence of ASA antibodies (20, 21), as these cause the release of calcium ions from the sarcoplasmic reticulum, thereby disrupting the excitation-contraction coupling mechanism of muscles, which may be related to myositis (22). The target antigens of ASA antibodies are native to skeletal muscle and cardiac muscle. Some scholars state that that they can be used as a clinical marker of MG with IIM (21), but further clinical studies with large samples are needed to confirm this.

In summary, when a patient has fluctuating muscle weakness, muscle soreness, and elevated muscle enzymes, it is necessary to be alert to the possibility of MG combined with IIM. The combination is often associated with thymoma and requires tumor-related examinations. The symptoms of most patients can be improved by plasma exchange and immunosuppressive therapy. When tumors are also found, they should be treated at the same time. The immune mechanism of MG-concomitant IIM when associated with thymoma is not yet clear and further research is needed.

## Data Availability

The original contributions presented in the study are included in the article/**Supplementary Material**. Further inquiries can be directed to the corresponding authors.
